# SERIES: eHealth in primary care. Part 3: eHealth education in primary care

**DOI:** 10.1080/13814788.2020.1797675

**Published:** 2020-08-06

**Authors:** Elisa J. F. Houwink, Marise J. Kasteleyn, Laurence Alpay, Christopher Pearce, Kerryn Butler-Henderson, Eline Meijer, Sanne van Kampen, Anke Versluis, Tobias N. Bonten, Jens H. van Dalfsen, Petra G. van Peet, Ybranda Koster, Beerend P. Hierck, Ilke Jeeninga, Sanne van Luenen, Rianne M. J. J. van der Kleij, Niels H. Chavannes, Anneke W. M. Kramer

**Affiliations:** a Department of Public Health and Primary Care (PHEG), Leiden University Medical Centre, Leiden, The Netherlands; b National eHealth Living Lab (NELL), Leiden, The Netherlands; c Medical Technology Research Group, Inholland University of Applied Science, Haarlem, The Netherlands; d Centre for Transformation in Digital Health, University of Melbourne, Melbourne, Australia; e Department of General Practice, Monash University, Melbourne, Australia; f College of Health and Medicine, University of Tasmania, Launceston, Australia; g Center for Innovation in Medical Education, Leiden University Medical Center, Leiden, The Netherlands; h Department of Anatomy and Embryology, Leiden University Medical Center, Leiden, The Netherlands; i Leiden Teachers' Academy, Leiden University, Leiden, The Netherlands; j Department of Obstetrics and Gynaecology, Erasmus Medical Center, Rotterdam, The Netherlands

**Keywords:** eHealth, education, primary care education, digital health, Continuing Professional Development (CPD), vocational training

## Abstract

**Background:**

Education is essential to the integration of eHealth into primary care, but eHealth is not yet embedded in medical education.

**Objectives:**

In this opinion article, we aim to support organisers of Continuing Professional Development (CPD) and teachers delivering medical vocational training by providing recommendations for eHealth education. First, we describe *what* is required to help primary care professionals and trainees learn about eHealth. Second, we elaborate on *how* eHealth education might be provided.

**Discussion:**

We consider four essential topics. First, an understanding of existing evidence-based eHealth applications and conditions for successful development and implementation. Second, required digital competencies of providers and patients. Third, how eHealth changes patient-provider and provider-provider relationships and finally, understanding the handling of digital data. Educational activities to address these topics include eLearning, blended learning, courses, simulation exercises, real-life practice, supervision and reflection, role modelling and community of practice learning. More specifically, a CanMEDS framework aimed at defining curriculum learning goals can support eHealth education by describing roles and required competencies. Alternatively, Kern’s conceptual model can be used to design eHealth training programmes that match the educational needs of the stakeholders using eHealth.

**Conclusion:**

Vocational and CPD training in General Practice needs to build on eHealth capabilities now. We strongly advise the incorporation of eHealth education into vocational training and CPD activities, rather than providing it as a separate single module. How learning goals and activities take shape and how competencies are evaluated clearly requires further practice, evaluation and study.

## Introduction

KEY MESSAGES

- eHealth education should be integrated into vocational training and continuous professional development programmes;
- Relevant topics are knowledge of applications, impact on stakeholder relationships, data utilisation and digital competence;
- eHealth training can be delivered in a variety of formats;
- CanMEDS and Kern’s model can be used to develop eHealth training programmes.

Innovative approaches are required to meet the demands presented by rapid changes in primary care, including issues such as an aging population, the increasing complexity of care such as increased knowledge of genetics (e.g. familial hypercholesterolemia) and pharmacogenetics, changing patient-provider relationships, a shortage of personnel, rapid technological developments and the recent developments around COVID-19 [1–3].

eHealth has a significant role in the present COVID-19 pandemic, and many general practitioners (GPs) are aware of the need to improve online communication with patients right now. Other examples include telemedicine and online mental health services [4,5].

Uptake of eHealth could be encouraged by broadening the focus of eHealth education to encompass the entire primary care team, including nurse practitioners, practice assistants, GPs involved in continuing professional development (CPD), and trainees undergoing vocational training [6]. The level and intensity of education depend on the training level, the needs of the specific target group and the context. An everyday case illustrates how the authors of this article foresee in the near future (2025), eHealth integration in daily practice and how educational strategies could support care providers and thus personalised medicine.

Dr. Smith (25 years old, female, general practitioner trainee) has an appointment with a patient (Mr. Jones, 45 years old). Mr Jones has a history of hypercholesterolemia, which has been hard to manage with statins due to numerous side effects. He has a positive family history for cardiovascular disease. His father and brother died at a young age from acute cardiac death due to coronary sclerosis. Blood pressure is acceptable (systolic blood pressure 140 mmHg and diastolic blood pressure 80 mmHg); however, over the last six months Mr. Jones has increasingly experienced problems due to dyspnoea when playing the trumpet. In light of the patient’s family history and increasing physical complaints, Dr. Smith is wondering how to proceed. In her practice, eHealth is commonly used in such situations during the successive phases of a patient’s journey from complaint to diagnosis and treatment. Nevertheless, as Dr. Smith is uncertain how to proceed and to what extent eHealth solutions are appropriate for this patient, she discusses the case with her primary care supervisor.

Although embedding of eHealth in educational programmes has acknowledged relevance, meaningful incorporation into medical education has been largely absent to date [7]. An Australian study has shown that because this topic is not addressed in medical education, the health care workforce is lacking the necessary competencies [8]. A comparable need to teach medical undergraduates through vocational training and beyond exists throughout Europe. However, despite several initiatives to incorporate eHealth into medical curricula, it is not yet common practice [9–11].

To operationalise eHealth in daily practice effectively, we propose that primary care providers should be supported, educated, and involved in all processes, from the development of effective eHealth solutions to their implementation in regular care. Addressing eHealth in medical education will not only result in an understanding of eHealth amongst doctors and the primary care team (e.g. practice assistants and nurse practitioners), it will consequently stimulate the uptake of eHealth in practice [12,13]. The impact of eHealth in daily practice is greater when it is integrated into usual care (blended care), which means enriching usual care with eHealth solutions rather than presenting eHealth as a stand-alone solution [14], and a greater impact is achieved when the whole practice team is involved, motivated and educated to organise blended care successfully [15–17].

The recent conceptualisation of eHealth made by Shaw et al., (Part 1 in this series), discerns three domains of eHealth, i.e. (i) ‘inform, monitor and track,’ (ii) ‘interaction,’ and iii) ‘data utilisation’ [2,18]. In addition, it is important to differentiate eHealth tools from eHealth services. eHealth tools comprise electronic health records (EHR), decision support systems and telehealth, whereas eHealth services include advice on eHealth policy and strategy or advice on eLearning programmes [7].

In this paper, we describe gaps in eHealth education and we try to support CPD developers and teachers providing medical vocational training by supplying suggestions for eHealth education and implementation. First, we describe what is required to help primary care professionals and trainees learn about eHealth and its integration in usual care. Second, we discuss how to do it, by giving advice on general educational activities directed at eHealth, by examining the design of a training programme using two distinct frameworks, and by providing several inspirational examples of best practices.

## eHealth education: WHAT?

Dr. Smith and her supervisor discuss the use of the EHR. The EHR delivers a reminder suggesting that Mr. Jones should be referred to the Cardiology department for a cardiac CT scan for coronary calcium, and that a genetic test for familial hypercholesterolemia (FH) should be considered. Dr. Smith proposes an online referral to the Department of Cardiology and wonders how to discuss preventive options with her patient, subsequently deciding to check how a FH test should be requested. She knows that evidence-based online information might be beneficial and therefore advises Mr. Jones to read information concerning FH and the possible implications for his children explicitly written for the general public. They decide to make an appointment to discuss the various options the following week.

The Mr. Jones scenario illustrates that there are several opportunities for eHealth to play a role in the delivery of care. Four topics relevant to the application of eHealth in education will be elaborated further, namely Knowledge of existing applications, Digital competence, Stakeholder relationships and roles and Data utilisation.

### Knowledge of existing applications

Health care professionals often lack knowledge regarding existing eHealth applications [19–21]. This knowledge includes (1) what apps exist for what purpose, (2) whether they are safe, evidence-based and effective, and (3) how apps should be implemented in daily practice. Health care providers can benefit from skills to appraise those aspects. In the case of Mr Jones, several eHealth applications could support Dr Smith. For example, regarding the ‘inform’ domain, a website aimed at GPs covers genetics (www.huisartsengenetica.nl) and includes information on hereditary forms of high cholesterol and referral criteria to the department of Clinical Genetics [22,23]. Regarding ‘monitor and track,’ eHealth has proven effective in improving cholesterol levels [24]. An example of ‘interaction’ is Shared Decision Making between patient and health care provider. However, Groenhof et al., did not report a clear clinical benefit of clinical decision support systems (CDSS) in terms of cardiovascular risk factor levels and target attainment, and they concluded that some features of CDSS seem more promising than others.

Dr. Smith understands how to use the EHR to record the patient’s relevant data. Furthermore, she can give advice on the best self-management application (app) designed for patients with high cholesterol and appropriate to the situation. Use of this app allows the patient’s health behaviour to be monitored, while at the same time educating the patient on the risks of high cholesterol. During the consultations, Dr. Smith can discuss the use of an app with Mr. Jones. Additionally, Dr. Smith can request a video consultation with the cardiologist to discuss the results of the CT scan. For research purposes, data on medical history, health behaviour, and health outcomes can be used for further studies of the risks of high cholesterol.

A clear description of the results of eHealth studies helps caregivers to select appropriate eHealth interventions. Use of CONSORT-EHEALTH guidelines is therefore recommended when reporting clinical trials results for eHealth interventions, while other guidelines such as the RECORD-PE are applicable for observational designs [25–29]. Knowledge of these guidelines could result in safe, evidence-based eHealth intervention choices and an understanding of the whole process of eHealth from development to implementation [2]. Required knowledge also includes information on the eHealth tools available to address specific medical problems effectively, and on the effectiveness of these tools [2].

### Digital competence

In all the domains identified by Shaw et al., a basic level of digital competence for health care providers is required to use eHealth in daily practice, in addition to sufficient access to ICT support [30,31]. There is an issue of potential generational differences, with trainees, younger doctors and younger patients being more familiar with digital solutions compared to older doctors and patients. Furthermore, from a provider perspective, digital competence includes digital skills and knowledge about aspects such as safety issues and ethics concerning online support [32]. Patient privacy must be guaranteed, and careful consideration should be given to determining the type of care that could be replaced or complemented with eHealth, in such a way that the quality of care remains unaffected. Regarding access to adequate ICT, it is important to be aware that available facilities may be outdated, both at the GP practice and for the patient. This may lead to difficulties in the use of eHealth since not all apps will work on older smartphones, laptops carrying outdated operating systems, or on desktop computers.

Another critical aspect of digital competence, especially from a patient perspective, is eHealth literacy. This includes traditional, computer, media, science, information and health literacy, in addition to numeracy [31]. It is important that eHealth is inclusive and that eHealth literacy receives the necessary attention to minimise the risk of inducing or increasing health inequalities [2]. In eHealth education, health care providers can learn how to deal with varying levels of eHealth literacy of their patients.

### Stakeholder’s relationships and roles

eHealth potentially influences patient-provider and provider-provider relationships. With regard to the ‘inform’ domain, the widespread availability of health information today may result in a patient being more informed about a particular medical topic than the physician, changing the role of the latter [33]. In addition, a general practitioner may advise a patient to seek further information tailored to that patient’s particular needs for example a video specially designed for patients with low health literacy. This strategy will improve a patient’s understanding and may, amongst other things, affect patient-physician interactions [34].

As regards the ‘monitor and track’ domain, eHealth applications facilitate patient self-management activities and promote personal control of decision-making on his or her treatment. Amongst other effects, these benefits have implications for the role of the physician.

Dr. Smith knows that both medication and lifestyle changes result in lower cholesterol levels. Using a shared-decision making tool, she can discuss the various options with Mr. Jones and help the patient select his preferred solution(s). In both cases, a self-management app can be used to track adherence and offer tailored support.

In addition to patient-provider roles, provider-provider roles and relationships may also change. For example, in the ‘interaction’ domain, a GP can more easily consult a medical specialist using teleconsultation but may miss ‘real life’ contact. Teleconsultation also shifts the role of the medical specialist from personal action to the provision of advice. In general, teleconsultation leads to a better-informed medical specialist who can provide more focussed advice, leading to justified advise from the GP and fewer referrals [35].

### Data utilisation

One of the eHealth domains described by Shaw is ‘data utilisation,’ which means gathering and filtering of relevant information, including (1) data from EHR system, (2) collection of data by patients using apps, often stored in cloud services, and (3) the connection and combination of data sources. Many stakeholders, including health care providers, can use these data sources for their own purpose, if they have had basic training in data sciences. Furthermore, the use of data could possibly be supported by the upcoming field of artificial intelligence (AI) [36]. Since AI and data sciences are relatively new terms within the medical profession, addressing these topics *via* eHealth education will have significant benefit for many health care providers [37,38]. The Royal College of General Practitioners (RCGP) recently published a report aimed at clarifying the position of AI [39]. Using information from the EHR, health care providers will be able to adjust treatment plans based on thorough symptom assessment, automated clinical coding, dermatological image recognition, triaging, and personalised self-management. A further possibility is proactive detection *via* analysis of patient records to identify undiagnosed conditions such as familial cancers or vulnerable groups such as frail older people. Algorithms developed for diagnostic or treatment purposes may help when making decisions on further diagnostics and personalised treatment plans [40].

A recent survey of AI mapping, conducted by NHS England showed that less than 10% of survey responders are currently applying AI. The RCGP stated that to utilise AI to its maximum potential, ‘*health care professionals will need to have access to education to learn new skills as AI users work differently to interpret AI outputs, with an understanding of its limitations and potential functions’* [37].

For example, pooling EHR data from many patients, combined with data from other sources, and facilitated by AI, may prove valuable to the design and refinement of (new) therapies. Nevertheless, it should be noted that using observational data might increase the risk of bias, due to confounding by indication, accuracy and reliability of routine data as there might be an overestimation of treatment effects [29]. Despite the promising opportunities, routine care data should therefore be used with caution.

Dr. Smith and Mr. Jones together decide to use an app aimed at lowering cholesterol levels. This app is part of a large real-life study on lifestyle and cholesterol. Dr. Smith informs the patient about this study and indicates that the patient can sign informed consent for this study digitally. Mr. Jones can also choose to share his data with his primary care professional. Over the following weeks Mr. Jones tracks his health behaviour and cholesterol, and the data is sent both to the researchers and his family physician. Dr. Smith uses this data during their next appointment to decide on further treatment.

## eHealth education – HOW?

Generally speaking, Dr. Smith was able to use eHealth solutions for this patient. However, it took her quite some time to figure out how this could best be achieved. Fortunately, she followed an online course provided by her vocational training institute and had a number of coaching sessions with her trainer regarding how her newly acquired knowledge could be applied in different cases, including exercises on how to use the app in daily practice. Nevertheless, she feels that acquiring these skills during her medical study would have been more efficient and sustainable.

### General suggestions for eHealth education

The reflections of Dr Smith detailed above give some insight into how eHealth competencies can be learnt and applied in practice: First, motivation combined with an awareness of the need to develop new competencies. Second, the opportunity to improve knowledge by following an eHealth course. Third, an opportunity to practice while supported by a supervisor. And, finally, reflection on what was learnt. These elements of learning are useful in relation to all four topics discussed in the ‘WHAT’ section, although the impact will vary depending on specific learning goals.

- Awareness of the need to learn more about eHealth competencies can be stimulated through motivational experiences from daily practice [41,42]. For example, the demand of safe video calling has increased tremendously as a result of the Covid-19 measures.
- A variety of opportunities exists to improve knowledge of eHealth, including vocational training day release courses, CPD courses, literature and eLearning, amongst others. As learning is subsequently enhanced and deepened by reflection, conversation and practice, blended learning is preferred over simple eLearning because it combines eLearning with discussion and performance [43].
- A simulation exercise with a standardised or virtual patient can be useful when practising eHealth, as this may improve familiarity with the necessary skills [44]. However, as eHealth is about the application of learning in daily practice, daily practice itself is an important educational activity [45]. If adequately supported, a supervisor is an important role model and coach during vocational training as one learns the most from real-life practice. As a coach, the supervisor can support the trainee through joint learning, by being available to discuss questions and dilemmas, and through promoting reflection and discussion.
- However, as a supervisor may not yet be skilled in eHealth, it is often beneficial to find an alternative role model through the GP residency programme with an affinity for eHealth and knowledge of daily practice. It may also be helpful to create a community of practice around eHealth together with other primary health care providers who have an interest in eHealth and have the opportunity to transform daily practice [46].

### Two possible frameworks to support the design of eHealth educational programmes

In this section, we describe how an eHealth educational programme can be deliberately designed, based on two examples: the CanMEDS framework and Kern’s model. The CanMEDS framework can be helpful when defining learning goals, while Kern’s model can be used to design effective eHealth training programmes [47,48].

**Figure 1.**
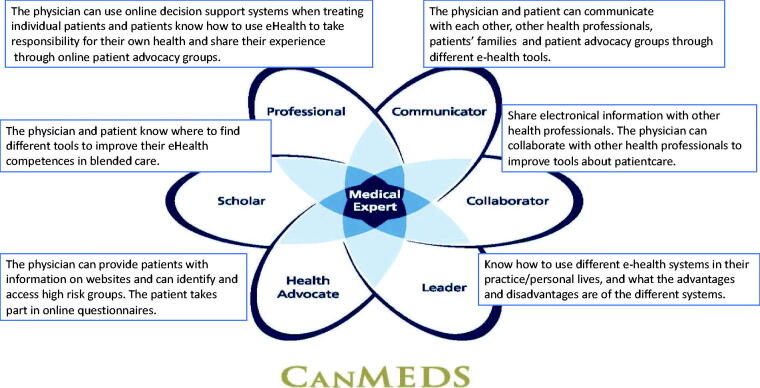
CanMEDS medical roles and related eHealth competencies [45].

CanMEDS framework to support the goals of eHealth education.A variety of frameworks are available that can support the goals of eHealth education. We chose the CanMEDS framework because it is used in many countries as an educational framework to guide medical education [49,50]. The CanMEDS framework highlights the various roles health care professionals can play and the varying competencies they should have. We have now added eHealth specific information to these roles (Figure 1) and have included the roles of patients.

Box 1. An example of a CanMEDS framework with roles and competencies adjusted for eHealth

Physician as a Communicator vs. Patient as a Communicator

Prior to the appointment, the physician sent a questionnaire in order to gather all relevant biomedical, psychosocial and family history data from the patient. The patient completed and returned the questionnaire. Both now have a communicator role. During the appointment the physician could use the results of the questionnaire to gather more detailed information (“*Inform, monitor and track*”), and a treatment plan was then developed based on shared decision making (“*Interaction*”).

In this scenario, eHealth is used in a similar fashion by both physician and patient, as both use it to share information to optimise the outcome of the appointment.

Physician as a Health advocate vs. Patient as a Scholar

As a health advocate, the physician has the task of providing information about websites covering, for example, healthy food and caloric intake, or of rebutting harmful medical misinformation circulating on social media (“*Inform*”).*^1^* The patient, in their role as Scholar, might want to learn more about a healthy lifestyle and visit certain websites. The websites, as a form of eHealth in this case, represent an easy way for the patient to gather information (“*Inform*”). Online consultation regarding these websites is a form of “interaction”. In this scenario the physician and patient have different roles, but both use the website for the purposes of encouraging a healthy lifestyle.

Physician as a Leader vs. Patient as a Leader

As a leader, one knows how to use different eHealth systems. For example, an online appointment system could help a physician manage his or her appointments. In this case, the physician is a Leader; the physician understands the advantages of the system and uses it in his work as a professional. The patient can also use this online appointment system. The patient uses it partly to manage his or her own care needs (“*Data utilization*”). In this case, the physician and patient both act as a leader by using an online appointment system in their care processes but with different goals regarding the eHealth tool. The physician uses it to manage appointments, whereas the patient uses it to manage and fulfil personal care needs.

Physician as a collaborator vs. Patient was a collaborator

Physicians can collaborate with other health professionals to share knowledge and improve tools related to patient care. Patients can share electronic health information with their health care professionals (“*Inform, monitor and track”; “Interaction”)*. In a personal health record, patients can track and manage their own health care data. Patients may also decide to share their data with specific health care providers. This data can be discussed during (tele)consultations.

Physician as a Professional vs. Patient as professional

The physician has an understanding of eHealth tools that can be used to support the treatment of individual patients. For example, they can use online decision support to determine treatment approaches based on existing data (*“Data utilisation).* Patients know how to take responsibility for their own health and can, for example, interact with peers via online platforms (*“Interaction”*). Furthermore, the patient could be involved in treatment decisions via shared decision tools.

In Box 1 we illustrate how an example of a CanMEDS framework, with roles extended to include the patient and an elaboration of corresponding competencies in line with the three eHealth domains of Shaw et al., as described in the WHAT section of this article, might be helpful in steering education in eHealth.

**Figure 2.**
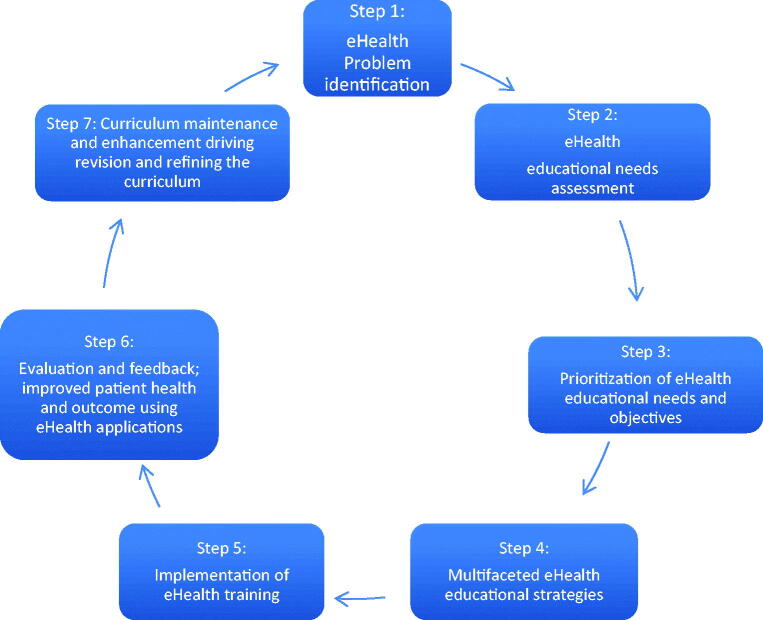
Conceptual model for the development, evaluation and maintenance of an eHealth education programme based on Kern [47,48].

Kern’s conceptual model for developing effective eHealth training programmes.Kern’s conceptual model can support the design of effective vocational and CPD training programmes. Education will only be effective in daily practice if it is aligned with the educational needs of the stakeholders who use eHealth in daily practice. In other words, it might be beneficial to adapt the way eHealth education is presented to a particular user and to tailor it to specific situations (Figure 2) [47,48].

Kern’s conceptual model can be used to develop, evaluate, and maintain eHealth vocational training and (online (e))CPD curricula [45]. Step 1 involves problem identification. In step 2, the educational gap is assessed, and educational needs are mapped. Since there are competing demands in vocational training and CPD, eHealth education is prioritised in step 3. As described in the ‘HOW’ section of this article, several eHealth educational strategies could be used in eHealth education (step 4). Step 5 then requires the adequate implementation of eHealth education. Based on the previous steps, step 6 then evaluates and gives feedback on the eHealth training and (e)CPD curricula. This step should be congruent with the previous steps described by Kern and should come to the ultimate goal of improved patient health and outcome using eHealth. Step 7 finally aims to maintain and enhance the curriculum in the rapidly changing field of eHealth; eHealth education should be revised and updated regularly.

### Best practices in eHealth education

Finally, we describe a few motivational examples of best practices. As demonstrated by the evidence discussed in this paper, the need for eHealth education has been advocated for the last decade, yet globally only sporadic examples can be found [51,52]. Nevertheless, these pioneering initiatives provide important insights into the development and implementation of eHealth education, with Table 1 summarising and reflecting on the usefulness of each initiative. For example, from the InHolland initiatives, it was experienced that introducing eHealth in the curriculum can be achieved in various ways and that the level of knowledge about eHealth from the teachers coupled with their engagement are important factors in that process [11,56].

**Table 1.** eHealth education initiatives and reflection on relevance.

Initiative	Description	Relevance/learning points
Clinical informatics education [53]	Based on the findings of an Australian project, suggestions are offered that may consistently improve the eHealth competencies of clinical health profession graduates and professionals.	Accrediting bodies and employers should identify and describe eHealth as a competence in their guidelines and job descriptions. All accrediting standards for health professionals should be reviewed to incorporate eHealth competencies. Ongoing professional registration should include eHealth professional development. Learning points: Further research is needed concerning (under)graduates studying for health profession degrees, including residency and CPD activities. Clarification is needed on the relevance and applicability of the eHealth skills acquired at a university or in a clinical workplace setting, and to effectively implement eHealth in daily primary care.
NFU module ‘create your own eHealth app [54]’	The project aims to develop an online education module concerning the careful development and evaluation of eHealth applications. This can be offered as an optional course in medicine (or related) programmes at Dutch University Medical Centres. During the module, the student learns how to develop a eHealth app. Minimally, this app should be an interactive prototype with the potential for further development to become an operational app with daily medical care functionality.	The application is an interactive prototype with potential for further development to an operational app aimed at educational practice. The student needs to formulate requirements and a (non)-functional design for the eHealth app. This design needs to be (partly) implemented in an interactive prototype. Learning points: Attention is paid to technological, organisational, financial, and legal aspects of eHealth apps and to user acceptance. The student learns to reflect on his or her eHealth application in the context of these factors.
InHolland Bachelor Nursing [13]	Students from the Bachelor programme Nursing receive an introduction on eHealth during the first semester. In that introduction, the students learn the basics of eHealth. They then work in interprofessional groups together with students studying technical subjects (e.g. electrotechnical engineering, informatics) with the goal of designing a technical solution for a health-related problem. Hackathons are used as a didactic approach to stimulating interaction. Following these first eHealth-related activities, throughout their studies the students are expected to include one substantiated technologically-based intervention in the care plans they formulate.	Students usually choose nursing for its ‘human side.’ The manner of introduction of eHealth stimulates a much-needed awareness of the use of technology in nursing. Embedding eHealth in the curriculum aligns with the new profiles of Nursing 2020. Learning Points: Challenges remain in: Organising around the differing schedules of interprofessional groups. Professionalisation of teachers to help them incorporate eHealth into their lessons.
InHolland Bachelor Dental Care [55]	The bachelor programme Dental Care contains many practical classes, which makes it more difficult to integrate eHealth into the existing curriculum. As a result, this bachelor subject has chosen to develop a separate educational track to introduce eHealth to the students so that they can begin to incorporate it into their practical classes.	The choice of a separate learning module on eHealth is a pragmatic solution, and the application of eHealth in dental care is relatively new. Learning points: Challenges remain in providing up-to-date contents for eHealth Dental Care.

## Discussion

This article is intended as a signal, with the goal of making the case for eHealth education to adequately prepare current and future health care providers and patients to incorporate eHealth in health care. Bringing together the *what* and *how* of eHealth education, leaves the question of *when* eHealth education should be implemented. As demonstrated through the evidence included in this paper, the need for eHealth education in health professional degrees has been advocated for the last decade, yet globally there has only been sporadic examples. Several initiatives provide greater knowledge of developing and implementing eHealth education, with Table 1 summarising and reflecting on the outcomes (i.e. enhanced knowledge, attitude change or behaviour change of each initiative).

However, more needs to be done. Globally, vocational and CPD training in General Practice needs to build on eHealth capabilities especially with the Covid-19 pandemic and its measures. First, to successfully integrate eHealth education into vocational and CPD training a change in culture is needed. This change should encompass an increasing sense of urgency regarding eHealth as a tool to meet existing challenges, combined with an understanding of the increasingly important role of eHealth as a component of health care. Consequently, there is an urgent need for eHealth education.

Second, we strongly advise the incorporation of eHealth education into vocational training and CPD activities, rather than providing it as a separate single module, as eHealth affects many aspects of health care. How learning goals and activities take shape and how competencies are evaluated clearly requires further practice, evaluation and study. Educational theories that are relevant to supporting eHealth learning in practice include transformative learning and expansive learning [57]. Appropriate research approaches to study and develop eHealth education include design-based and action research [58].

Barriers to and possible disadvantages of eHealth also need to be considered. For example, eHealth illiteracy is a potential barrier both for the patient and the provider. Another barrier is a reluctance to use eHealth, both from patient’s and the physician’s perspective [53]. This might be related to negative experiences, doubts regarding the evidence, or time constraints [54]. On the other hand, positive experiences might remove this reluctance. We have already mentioned the COVID-19 crisis, and current experiences may have a positive impact on the understanding of and use of eHealth. Nevertheless, eHealth is not a goal on itself and we must emphasise that eHealth is simply a tool to manage existing problems. Furthermore, the sustainability of eHealth is an important challenge, as sustainability is known to be affected by financing strategies [55,59]. A recent study showed that eHealth sustainability could be supported by integrating sociotechnical aspects in financing models. Agreements between public and private sectors, and between public organisations and the general population, are recommended [60]. These barriers to eHealth applications legitimately require attention and dealing with them will support the use of eHealth in practice.

## Conclusion

This paper is a first step towards awarding eHealth education the attention it deserves. We have established that the central issue in eHealth is learning itself, and provide ideas regarding appropriate educational activities at three levels: easy to implement general suggestions, two training programme design frameworks, and examples of best practices. We strongly advise integrating eHealth into vocational training and CPD, together with thought regarding possible barriers to uptake. The study and further development of eHealth education should be a priority for all future-focussed health care providers.
